# An evaluation of author productivity in international radiography journals 2004–2011

**DOI:** 10.1002/jmrs.21

**Published:** 2013-08-26

**Authors:** Beverly A Snaith

**Affiliations:** Radiology Department, Mid Yorkshire Hospitals NHS Trust, Pinderfields HospitalAberford Road, UK

**Keywords:** Author productivity, bibliometrics, medical radiation sciences, publication, radiography

## Abstract

**Introduction:**

Radiography, the allied health profession, has changed beyond recognition over the last century; however, in academic terms radiography is a relatively young profession. It is therefore still establishing its professional knowledge base. This article uses peer-review author productivity distribution to evaluate its scholarly maturity.

**Methods:**

Four peer-reviewed journals in medical radiation sciences were examined over an 8-year period (2004–2011) and author productivity was compared to Lotka's law. Further analysis of the most prolific authors provided an evaluation of their characteristics.

**Results:**

The 1306 unique authors contributed 835 articles during the study period. Of these, 1012 (77.5%) contributed only one article to the journals studied, with an inverse power relationship of author productivity. At the 0.1 level of significance, radiography does not fit Lotka's law (*n* = −2.334; *c* = 0.712; *D*_max_ = 0.0627; Critical threshold = 0.0337). There was a significant correlation between the most prolific authors and collaboration (*P* = 0.002), although variation was noted in author discipline and location.

**Conclusions:**

The results of this study add to the discussion of radiography scholarship and demonstrate that the radiography authors have similar productivity distribution to other professions, but do not follow Lotka's law.

## Introduction

In order for the profession of radiography to remain evidence based, it requires both individuals and teams to share their knowledge and discoveries through publication and presentation at conferences. The dissemination of research findings ensures that diagnosis and treatment, together with the underpinning education, remain at the forefront of professional knowledge and represents the final stage in the research process.

The radiography profession has changed beyond recognition over the last century, with radiographers evolving from former assistant roles through self-discovery to emerge as an autonomous evidence-based profession. Many countries have now developed career progression models resulting in the blurring of professional boundaries and the sharing of a number of interpretive and/or procedural tasks.^1^,^2^ In academic terms, radiography is still relatively young, only entering the university sector in the 1990s,^3^,^4^ although education and status still vary internationally.^1^,^4^,^5^ It remains, however, unclear whether these clinical and academic developments have improved research and publication rates as a result of, or unrelated to, the promotion of advanced and consultant radiographer (and senior academic or professorial) roles.

In relation to the research base of a profession, examination of author productivity has been used as proxy to demonstrate scholarly maturity.^5^–^7^ Diagnostic radiography author productivity has only been reviewed on one longitudinal single-journal study,^8^ but studies from other disciplines have concluded that the most productive authors will contribute disproportionately to the evidence base and is illustrated by Baker et al.^9^ in their review of sport psychology which found that just 3% of authors provided 24% of publications. To assess author productivity, a comparison with Lotka's law is the most widely cited method and has been tested by numerous studies in many disciplines.^6^,^7^,^10^ Alfred Lotka published his seminal research in 1926 and found that authorship within a mature profession followed an inverse square distribution with the number of authors writing *x* articles equating to 1/*x*^2^ of those authoring just one article.^6^,^7^ In practical terms, Lotka's law implies that for every 100 authors writing one article, only 25 will write two, 11 will write three, etc. When plotted graphically, the slope of the line of author productivity has been reported as −2 (inverse square). Later research suggests that it actually may lie between −1.2 and −3.8 and still allow correlation with the Lotka distribution, but should be more appropriately termed an inverse power law.^7^

Comparison with Lotka's law can evaluate a profession, speciality, or journal, but the most well-known publication metrics are “impact factor” which evidences the impact of a journal through citation analysis,^11^ and the “*h*-index,” named after its creator Jorge Hirsch, designed to measure an author's productivity and impact through their citations.^11^–^13^ These measures are, however, inconsistent across disciplines due to differences in citation patterns.^11^ Author productivity on a macro (professional) or micro (individual) level has not previously been examined in radiography, and this study builds on a study of international radiography peer-reviewed journals^14^ through a systematic review of author outputs.

## Objectives

To establish the level of author productivity within international radiography journals and analyse the characteristics of prolific authors to establish any underlying trends.

## Methods

This study was a secondary analysis of bibliometric data^15^ compiled from four English-language radiography journals covering both the diagnostic and therapeutic disciplines. Other journals were identified which met some of the criteria, but were focused on a single discipline or modality, for example, radiation therapy, ultrasound, magnetic resonance imaging, and were therefore excluded. The sample was therefore comprised of publications reflecting the medical radiation sciences profession within a single journal and provided a global picture – the *Journal of Medical Imaging and Radiation Science* (Canada), *Radiography* (United Kingdom [UK]), *The Radiographer* (Australia – now replaced by the trans-Tasman *Journal of Medical Radiation Sciences*), and *The South African Radiographer* (South Africa). The data spanned an 8-year period from 2004 to 2011 and included original research, review articles, guest editorials, case reports, and correspondence. No ethical approval was required for this study.

Controversy exists in bibliometric research as to whether analysis of author productivity should include only the “senior” author or all contributors,^7^,^10^ and whether authors should be wholly or fractionally counted.^9^,^15^ Convention is that the most senior author is listed last as they are often the project supervisor, although the radiography literature does not wholly support this premise, and therefore data for all authors were included. Although this means that the opportunity of direct comparison with some of the previous examinations of Lotka's law is not possible, it does ensure that any comparisons are consistently using the same data set. The least squares method was used to identify the productivity gradient where the number of data entries, *N*, is 15, *X* is the logarithm of the number of articles published (1, 2, 3, …, 19), and *Y* is the logarithm of the number of authors^10^:


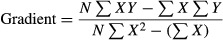


Author frequency and goodness of fit were evaluated using the one-sample Kolmogorov–Smirnov (K–S) test for ranked data. The K–S test requires calculation of the fraction of authors expected to publish one article (*c*) within the sample and also uses *P* to represent the number of articles published^15^:


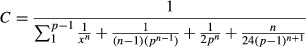


The most productive author data were also examined in terms of demographics (country, discipline, and subject) and collaboration. The total articles published, including those outside the studied journals, and author *h*-indices for the same period were identified from Scopus (Elsevier 2013). Correlation between author productivity, collaboration, and *h*-index was calculated using Spearman rank correlation coefficient (SPSS version 16.0; Chicago).

## Results

**Figure 1:**
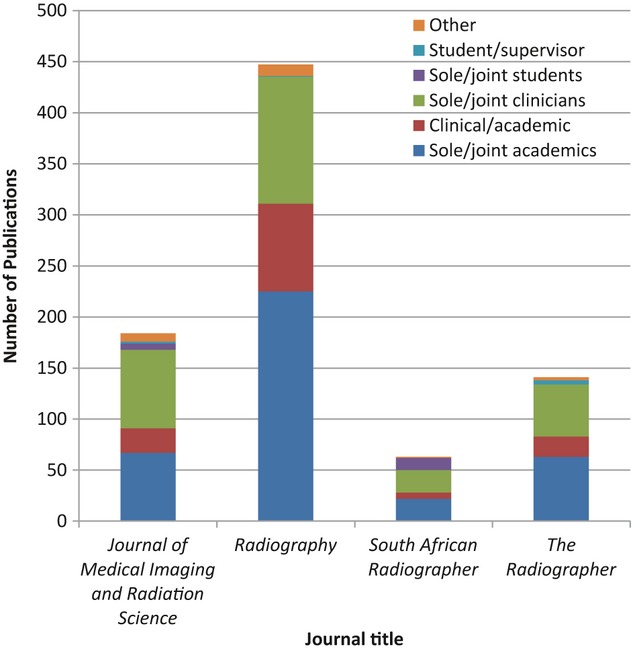
Author status across the four radiography journals.

A total of 835 articles were identified which met the inclusion criteria with a total of 1999 authors listed. These names actually represented 1306 unique authors who were predominantly academics or academic collaborators (*n* = 519/835; 62.2%), but this was seen to vary across journals (Fig. 1). As author status is not consistently reported, the number of student articles cannot be accurately calculated, and only those articles which explicitly described an author as a student have been classified as such. The *South African Radiographer* (SAR) published the greatest number of student articles, 19% of total number of articles (*n* = 12/63), all case reports submitted by undergraduates, either alone or in groups.

Despite the volume of articles reviewed, the majority of authors (using whole counting – single count per author per article, rather than part) contributed to just one article (*n* = 1012/1306; 77.5%), with only 9.4% of authors publishing more than twice over the 8-year period (*n* = 123/1306); however, one individual authored 19 articles (Table 1).

**Table 1:** Distribution of radiography author productivity (2004–2011)

Author publication productivity	Number of authors	Accumulated publications (%)	Accumulated authors (%)
19	1	19 (0.95)	1 (0.08)
14	2	47 (2.35)	3 (0.23)
13	3	86 (4.30)	6 (0.46)
12	1	98 (4.90)	7 (0.54)
11	4	142 (7.10)	11 (0.84)
10	3	172 (8.60)	14 (1.07)
9	4	208 (10.41)	18 (1.38)
8	5	248 (12.41)	23 (1.76)
7	5	283 (14.16)	28 (2.14)
6	13	361 (18.06)	41 (3.14)
5	7	396 (19.81)	48 (3.68)
4	24	492 (24.61)	72 (5.51)
3	51	645 (32.27)	123 (9.42)
2	171	987 (49.37)	294 (22.51)
1	1012	1999 (100)	1306 (100)

Using the least squares method the gradient of the slope of author productivity (*n*) was calculated as −2.334. As the value of *n* (−2.3) lies within the limits previously described it implies that radiography authorship follows an inverse power distribution, with most authors publishing only one article and significantly smaller numbers contributing higher publication levels.

Based upon Lotka's calculations and using the exponent specific to radiography (−2.3) the results demonstrate that *c* = 0.712, suggesting that an expected 71.2% of authors would publish one article, whereas the observed number was actually 77.5%. The author data can be further assessed in a comparison of observed and predicted authorship values in the K–S goodness-of-fit test (Table 2).

**Table 2:** The K–S test for radiography authorship

Author publication productivity	Observed value	Accumulated observed value Sn(*x*)	Predicted value	Accumulated predicted value Fo(*x*)	Absolute value Fo(*x*)-Sn(*x*)
1	0.7749	0.7749	0.7122	0.7122	0.0627^1^
2	0.1309	0.9058	0.1412	0.8534	0.0524
3	0.0391	0.9449	0.0548	0.9083	0.0366
4	0.0184	0.9632	0.0280	0.9363	0.0270
5	0.0054	0.9686	0.0166	0.9529	0.0157
6	0.0100	0.9686	0.0109	0.9638	0.0148
7	0.0038	0.9824	0.0076	0.9714	0.0110
8	0.0038	0.9862	0.0056	0.9769	0.0093
9	0.0031	0.9893	0.0042	0.9812	0.0071
10	0.0023	0.9916	0.0033	0.9845	0.0075
11	0.0031	0.9946	0.0026	0.9871	0.0062
12	0.0008	0.9954	0.0022	0.9892	0.0067
13	0.0023	0.9977	0.0018	0.9910	0.0067
14	0.0015	0.9992	0.0015	0.9925	0.0067
19	0.0008	1.00000	0.0007	0.9933	0.0067

1 Maximum deviation (*D*_max_).

The results show that the whole author count does not fit with Lotka's law at the 0.1 level of significance as the variation between expected and observed authorship, the Dmax, does not reach the critical threshold (*c* = 0.712; *D*_max_ = 0.0627; Critical threshold = 0. 0337).

The 1306 authors were ordered by publication productivity and the 25 most prolific authors were identified.^16^ As this cut-off was within a group of authors with seven publications, the 23 authors who wrote more than eight articles were selected for further evaluation (*n* = 23/1306; 1.8%). These 23 contributed to 247 articles (range 8–19) with 83.0% of them collaborative (*n* = 203/247), although the level of collaboration varied between authors (Table 3). As 38 of these collaborative articles were with other prolific authors, the number of unique articles authored by this cohort was 167, representing 20% of all the publications reviewed within this study (*n* = 167/835).

**Table 3:** Details of prolific author publications

	Productivity		Collaboration			
				
Author	Articles	Rank	Articles (%)	Rank	Total articles^1^	*H*-index^2^
Brennan, Patrick	19	1	19 (100)	1	75	8
Bolderston, Amanda	14	2	11 (78.6)	5	17	5
Hogg, Peter	14	2	14 (100)	2	17	4
Marshall, Gill	13	4	8 (61.5)	14	14	4
McEntee, Mark	13	4	11 (84.6)	5	38	5
Warren-Forward, Helen	13	4	13 (100)	3	20	5
Hardy, Maryann	12	7	12 (100)	4	13	5
Bentley, H Brian	11	8	1 (9.1)	23	9	0
French, John	11	8	5 (45.5)	21	11	5
Middleton, Mark	11	8	11 (100)	5	8	2
Poulos, Ann	11	8	9 (81.8)	8	16	6
Cox, Jennifer	10	12	9 (90.0)	8	21	5
Reeves, Pauline	10	12	6 (60.0)	19	13	3
Snaith, Beverly	10	12	9 (90.0)	8	11	5
Currie, Geoffrey	9	15	9 (100)	8	48	5
Davidson, Robert	9	15	9 (100)	8	11	4
Halkett, Georgia	9	15	9 (100)	8	34	8
Smith, Tony	9	15	4 (44.4)	22	18	5
Kurmis, Andrew	8	19	8 (100)	14	20	4
Nightingale, Julie	8	19	7 (87.5)	18	10	3
Palmer, Cathryne	8	19	8 (100)	14	10	4
Reed, Warren	8	19	6 (75.0)	19	9	3
Wheat, Janelle	8	19	8 (100)	14	45	5

^1,2^Scopus – limited to articles 2004–2011(accessed 27 February 2013).

The productivity of these authors was subsequently ranked in relation to individual and collaborative articles (Table 3) and to identify whether these authors were prolific only in radiography or they had wider influence, the Scopus publication figure and *h*-index for the same period (2004–2011) were also identified.

These authors not only contributed the most articles to the journals examined, they also published widely, with a mean of 21 articles over the 8 years (range 8–75). Spearman rank coefficient of the ranked data demonstrated significant correlation between productivity and collaboration (ρ = 0.6; *P* = 0.002).

Analysis of the prolific author data shows the prolific authors to all be members of the medical radiation sciences profession, with the majority from the diagnostic discipline (16/23; 69.6%). Only three countries are represented (UK, Australia, and Canada), but perhaps unsurprisingly these correspond to three of the four journal publishing countries; however, two of the authors relocated within the study period, having previously published from addresses in Eire. If the more recent location is used then Australian authors predominate (13/23; 56.5%).

Although the UK journal *Radiography* published the most articles over the study period (*n* = 447/835; 53.5%), only seven of the prolific authors are based in the UK (*n* = 7/23; 30.4%). In relation to article subject, the authors wrote on a range of topics, but the most frequently occurring research interests/themes were role development and image perception.

## Discussion

Only limited investigation of radiography publication practices have been previously undertaken, including a mapping of single UK and US journals.^8^,^14^,^17^,^18^ However, these have demonstrated the increasing appetite for the use of and contribution to the health evidence base. The four journals evaluated in this study are not indexed on Scopus or any other single database and therefore some omissions of data are evident.

Radiography is a relatively young profession in terms of scholarly activity,^4^,^5^ which probably explains why the examination of the contribution of radiography authors to the evidence base has been limited. However, publications can be used as an indicator of research activity^4^,^19^ and such outputs can measure both research quantity and quality,^19^,^20^ but not impact.^13^ As radiographers publish in both disciplinary and wider journals, actual assessment of total radiography research activity is difficult as affiliation of clinical radiographers is common to a radiology or oncology department and therefore indistinguishable from medical authors. The examination of profession-specific journals can assist in our understanding of scholarship, and this study suggests that radiography, as represented by the four international journals, does not match the distribution of author productivity expected by Lotka's law when whole author count is used. It appears disappointing that only 22.5% of senior authors published more than one article, although this is broadly in line with the expected level established from the calculations and is consistent with other studies.^21^–^23^ More important is to recognize that across four journals and 8 years, 20% of the publications in this study (167 unique articles) were written by only 3% of the journal contributors. This skewed distribution is similar to the results of Baker et al.^9^ and demonstrates the potential level of influence that a relatively small number of individuals may have on the whole profession. The significant contribution of a small number of authors to an individual evidence base is a common theme in the wider literature, and there is ongoing debate as to whether a discipline is influenced more by the limited volume of work produced by a broad body of scholars or the larger contribution of an “eminent few.”^9^,^22^ Research previously investigating the factors which influence the prolific “few” proposed cumulative advantage and the superstar phenomenon to explain success factors including motivation, creativity, training, and work habits.^22^

Despite the relatively small number of radiographers working in Australia, they represented the highest number of prolific authors, slightly skewed by migration, but still the most productive. This suggests that the radiography research culture within Australia is positive. There is also an overrepresentation of radiation therapists within the top 23, again predominantly Australian and Canadian authors. The lack of UK therapists within the most prolific author sample may be influenced by the presence of a separate successful UK therapy-focused journal, the *Journal of Radiotherapy in Practice*, although no data of its author profile are available. Although the UK journal *Radiography* has the longest history as a peer-reviewed journal and greatest publication numbers, less than one-third of the most prolific authors are UK based, but the journal has been shown to publish a significant proportion of international articles.^14^,^17^

Rather than the most prolific authors only submitting to the journals within this study, the data indicate their ongoing contribution to wider peer-reviewed journals, with an average of 21 publications and *h*-index of 4.5. No previous study of radiographer *h-*indices has been undertaken, but this figure is lower than that of US academic oncologists,^24^ but within the range of radiologists,^25^ suggesting that the most successful radiographers are working at an equivalent level of medical peers. Obviously citations are dependent on subject and potential audience size, illustrated by the low *h*-index of Bentley, whose articles are predominantly historical commentaries. There is currently no specific benchmark for the *h*-index, but appropriate disciplines levels need to be established as interdisciplinary comparison may be unfair.^12^,^26^ Such benchmarking will, however, need to be systematic in its data extraction as the results of this study confirm the issues with retrieving citation data due to the indexing inconsistencies. This is illustrated in Table 3, with one author (Middleton) seen to have a lower total publication record on Scopus than in this study and therefore the overall figures are likely to provide an underestimation of author activity, although Scopus has been suggested as the most inclusive database.^13^,^27^

It is interesting to note that a large number of the most prolific authors are involved in the leadership of the journals studied, including current or previous editors-in-chief, including French (*JMIRS*), Bentley and Hogg (*Radiography*), and a further 12 are members of one or more of the journal editorial boards. It has previously been suggested that editorial appointments are an acknowledgement of the most productive or influential researchers and that a consequence of such roles may result in these individuals being more successful.^23^

Academics as solo or collaborative authors were the most productive, not only within the top 23, but also the whole author cohort, producing 62.2% of all articles, a similar proportion to previous studies.^17^,^18^ Despite the international drive for clinical research development, including a 10-year history of formalized advanced and consultant radiographer roles in the UK, the results do not yet appear to be evident with only a small number of productive clinicians; however, such strategies do require long-term development. Interestingly, Canada showed a higher number of clinical authors than academics, which may be as a result of their drive to develop clinical research.^4^ Strategies to develop writing skills among potential authors are important and opportunities do exist for mentorship by experienced authors, although greater emphasis is probably required on those engaged in academic studies.

The inconsistency in recording of author status means that student work cannot be accurately captured, but some of the clinical and/or academic categorized authors may also have written work completed as an undergraduate or postgraduate student. It is therefore not clear what proportion of coauthorship is related to supervision of student dissertations. This is an area which has been identified as an opportunity to develop writing skills and is encouraged for both academics and students alike.^28^

This study has demonstrated significant correlation between prolific authors' productivity and coauthorship, confirming the findings of previous research.^16^ The coauthor may be a colleague, research collaborator, or academic supervisor, but the addition of another individual may provide the impetus to complete the transition from idea or data to published article. Collaboration has been shown to positively influence author productivity and also increase citations^29^ and therefore has potential to increase an individual's *h*-index.

## Conclusion

This publication analysis has provided a limited overview of research activity in radiography, and although the profession does not appear at this time to correlate with Lotka's law, it demonstrates a pattern of productivity in line with other professions, with a significant number of one-time authors and small number of recurring author names. The international profile of prolific authors evidences an evolving research base and confirms that collaboration increases individual productivity.

Bibliometric evaluation is a relatively new field to radiographers; however, ongoing debate about research productivity and the radiography evidence base requires such methods to evidence the impact of current and future research strategies. Further debate about the anticipated level of scholarly activity, such as research and publication, by both academic and/or clinical radiographers is needed.
